# Supplementary material for: In Situ Cell Signalling of the Hippo-YAP/TAZ Pathway in Reaction to Complex Dynamic Loading in an Intervertebral Disc Organ Culture
Source: Int J Mol Sci. 2021 Dec 20;22(24):13641. doi: 10.3390/ijms222413641 (PMC8707270; doi:10.3390/ijms222413641)
Supplement: Supplementary file 1 [file ijms-22-13641-s001.zip › Manuscript+Figures/Figure_S1 Figure Legends.docx]

**Figure Legends for Figure S1**

**Close-up immunofluorescence pictures of YAP at 40x magnification**

**Figure S1.** Immunofluorescent pictures of the nucleus pulposus (NP) and in the annulus fibrosus (AF). The sections were stained with DAPI (blue) and anti-YAP (green) and are shown individually as well as merged. (a) NP cells at day 0, (b) NP cells after static load, (c) NP cells after low-stress load, (d) NP cells after intermediate-stress load, (e) NP cells after high-stress load, (f) AF cells at day 0, (g) AF cells after static load, (h) AF cells after low-stress load, (i) AF cells after intermediate-stress load, (j) AF cells after high-stress load. Scale bar = 50 µm.
