# Supplementary figures and images for: In Situ Cell Signalling of the Hippo-YAP/TAZ Pathway in Reaction to Complex Dynamic Loading in an Intervertebral Disc Organ Culture
Source: Int J Mol Sci. 2021 Dec 20;22(24):13641. doi: 10.3390/ijms222413641 (PMC8707270; doi:10.3390/ijms222413641)

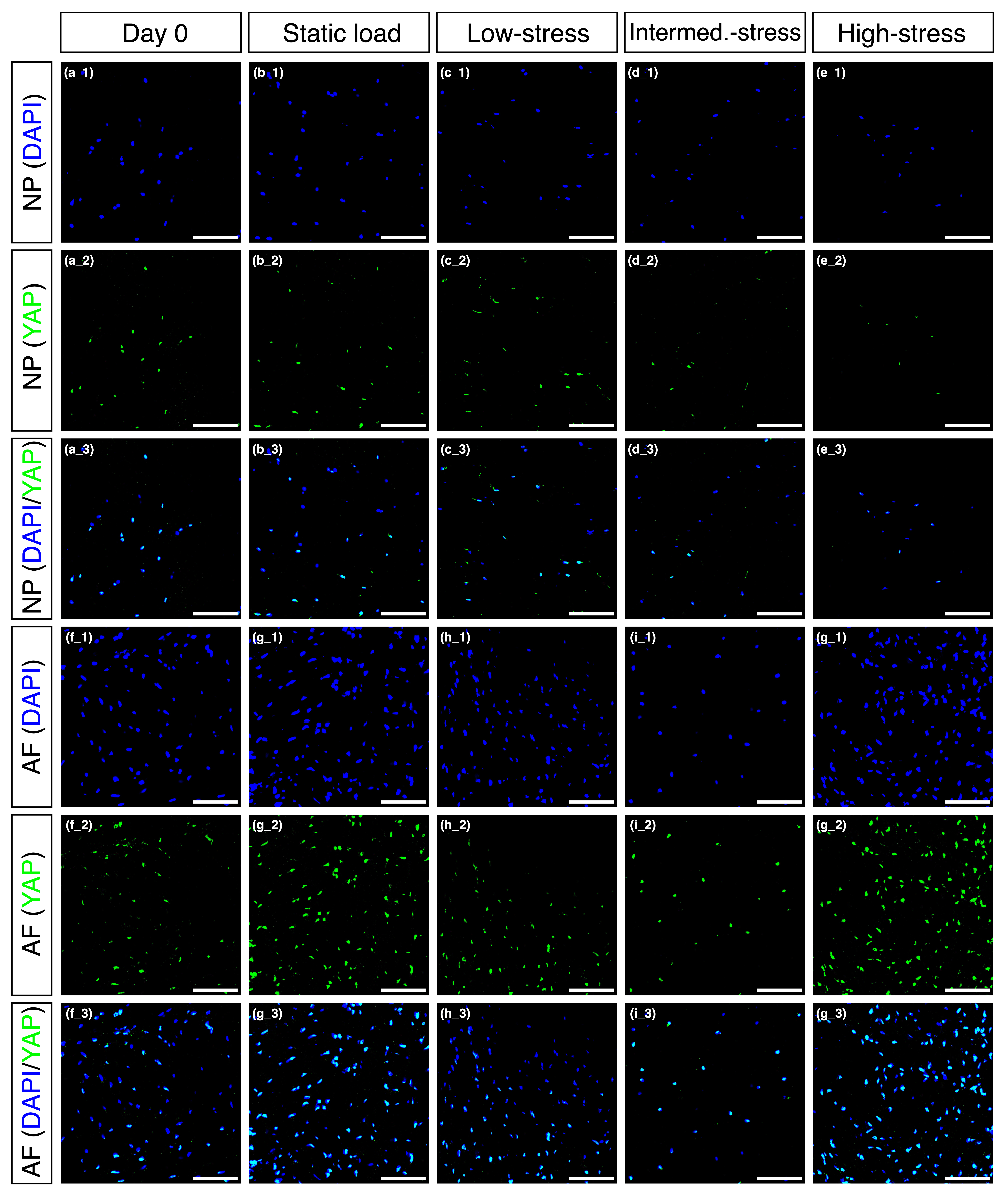

Supplement: Supplementary file 1 [file ijms-22-13641-s001.zip › Manuscript+Figures/Figure_4.jpg]

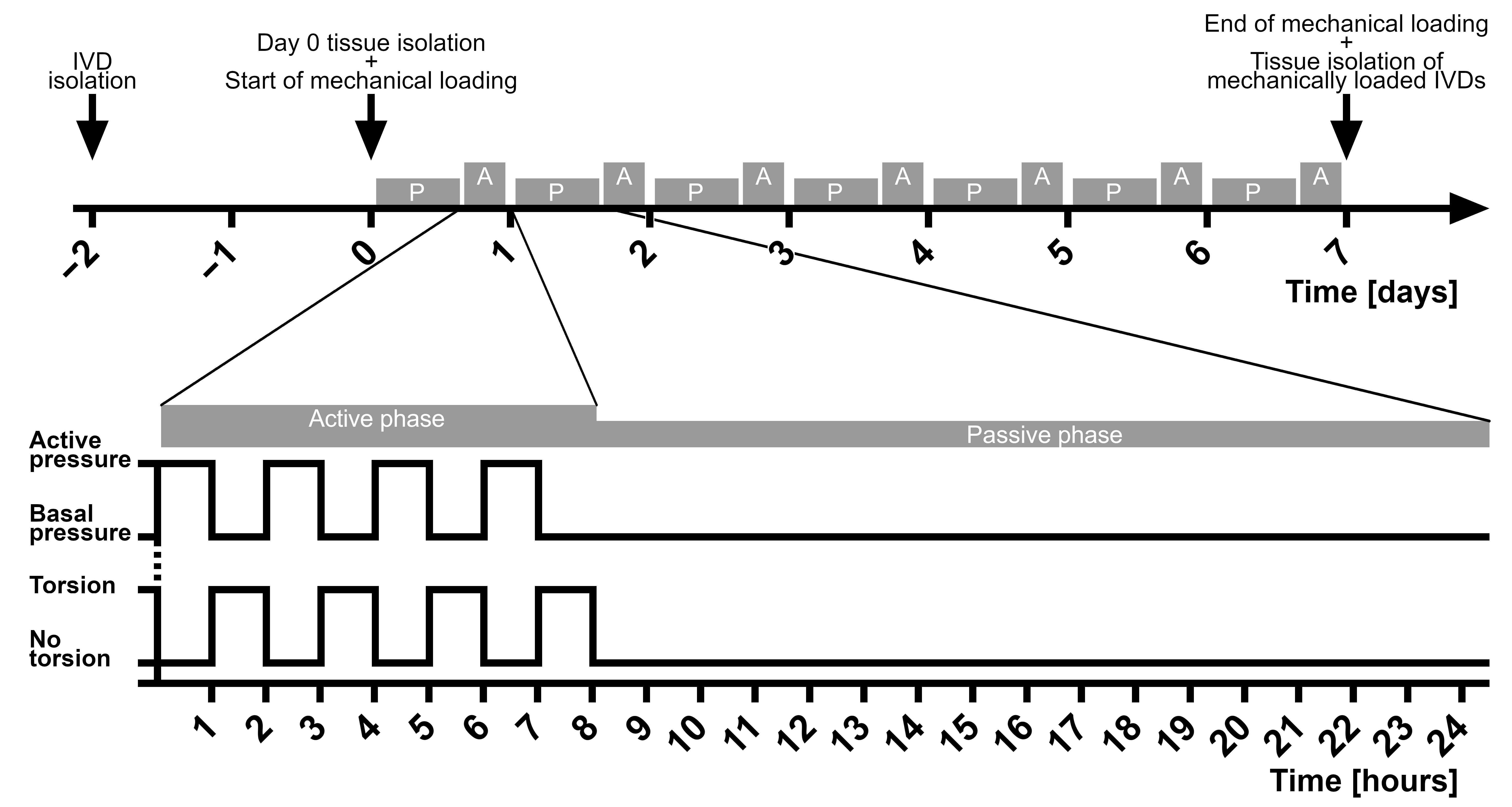

Supplement: Supplementary file 1 [file ijms-22-13641-s001.zip › Manuscript+Figures/Figure_5.jpg]

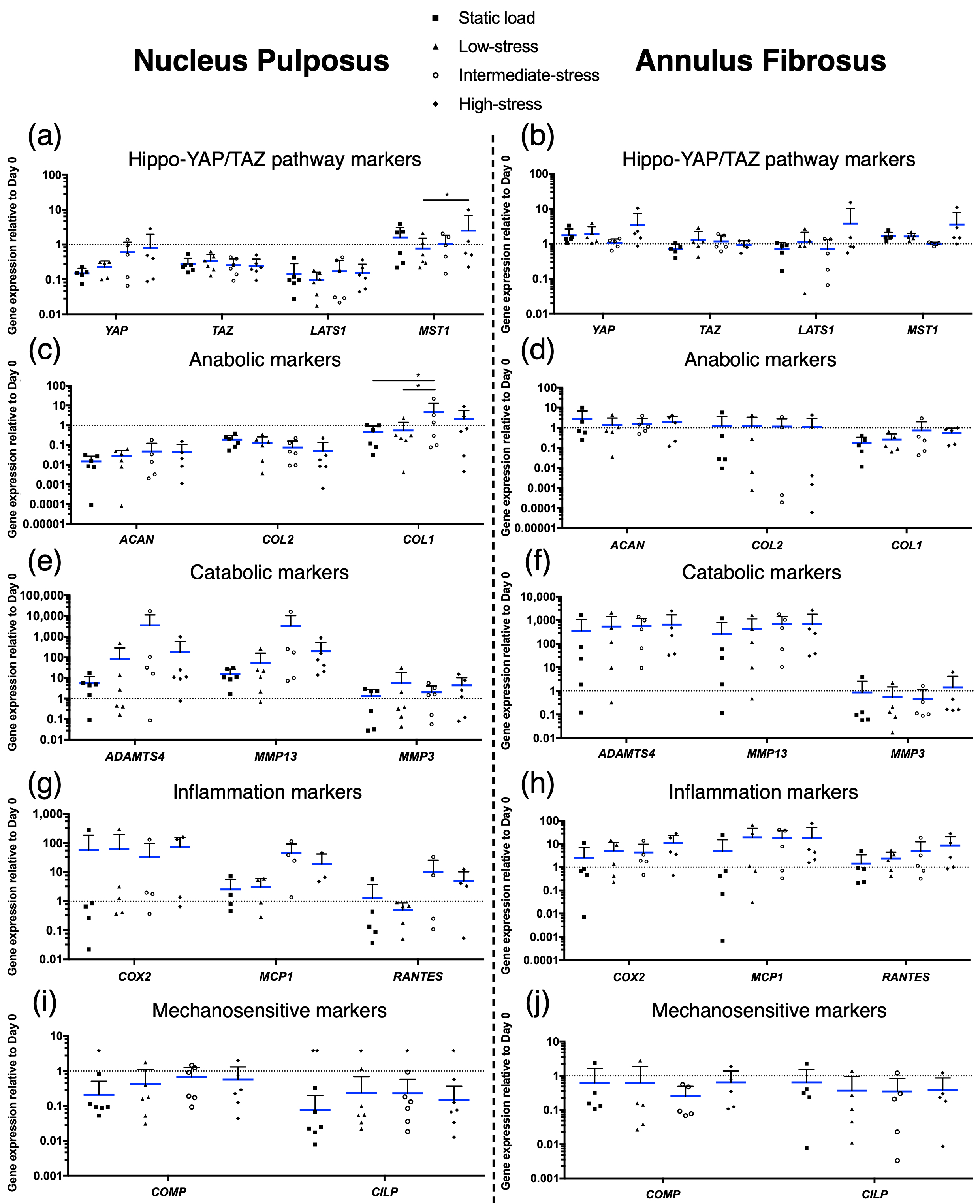

Supplement: Supplementary file 1 [file ijms-22-13641-s001.zip › Manuscript+Figures/Figure_3.jpg]

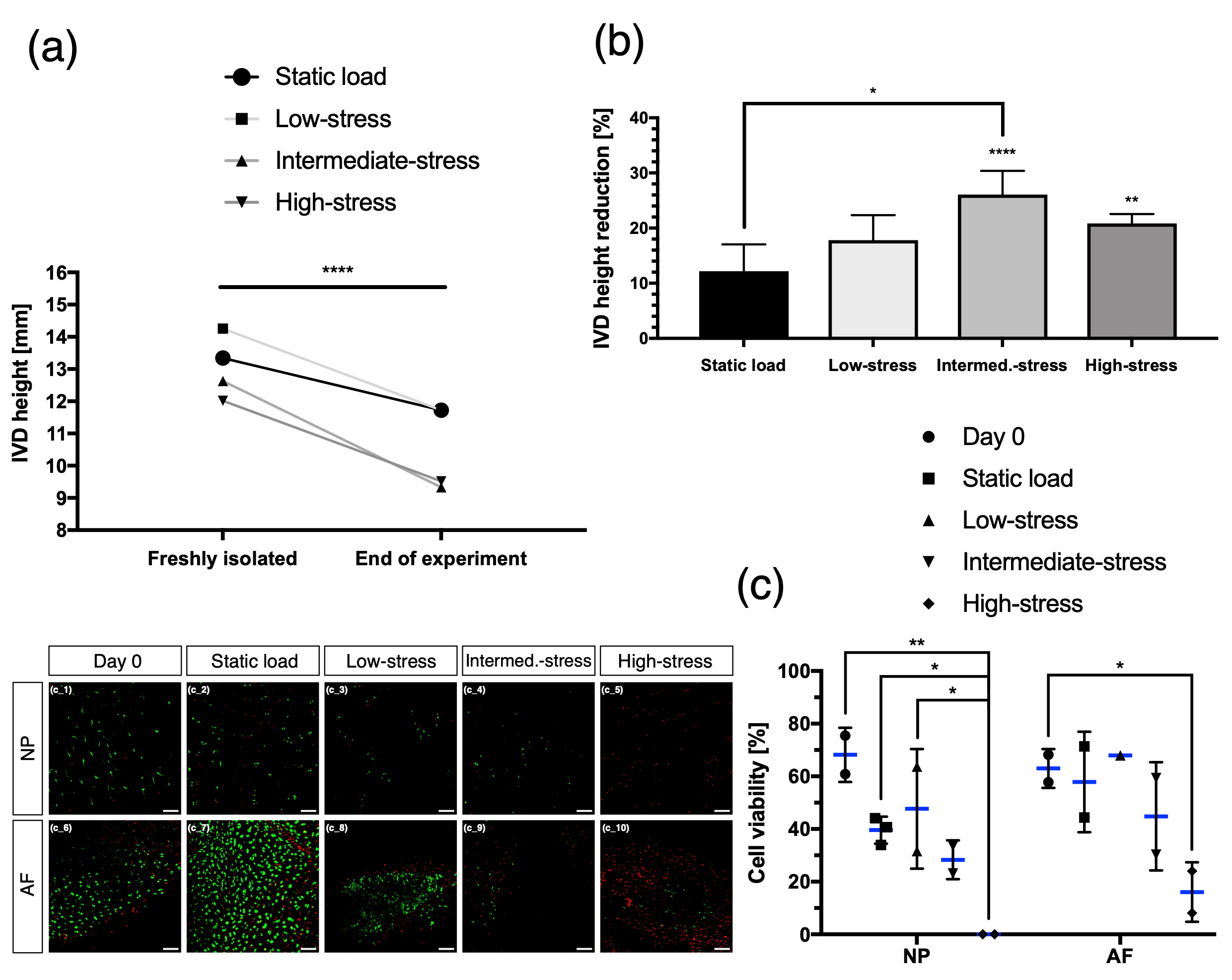

Supplement: Supplementary file 1 [file ijms-22-13641-s001.zip › Manuscript+Figures/Figure_1.jpg]

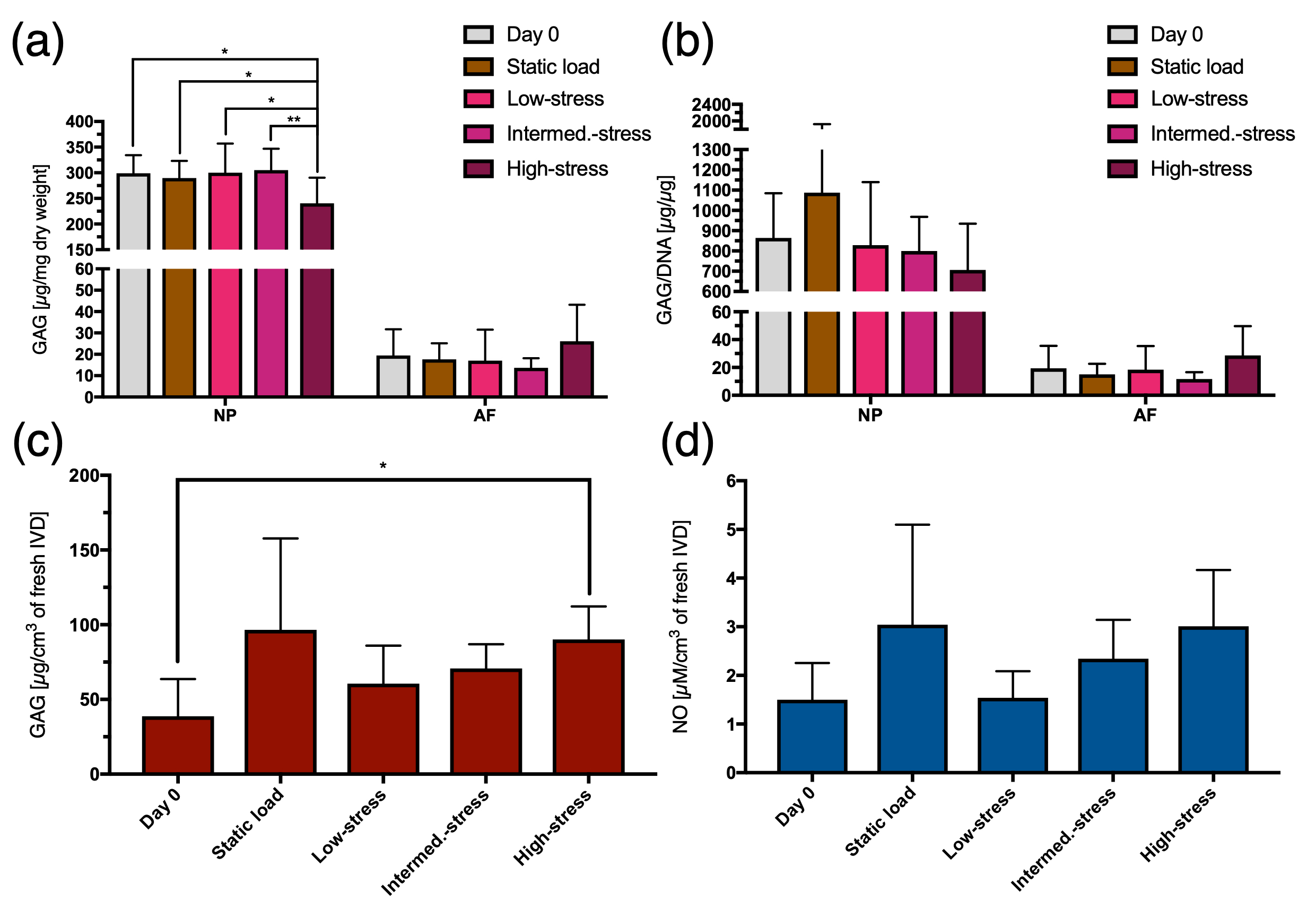

Supplement: Supplementary file 1 [file ijms-22-13641-s001.zip › Manuscript+Figures/Figure_2revised.jpg]

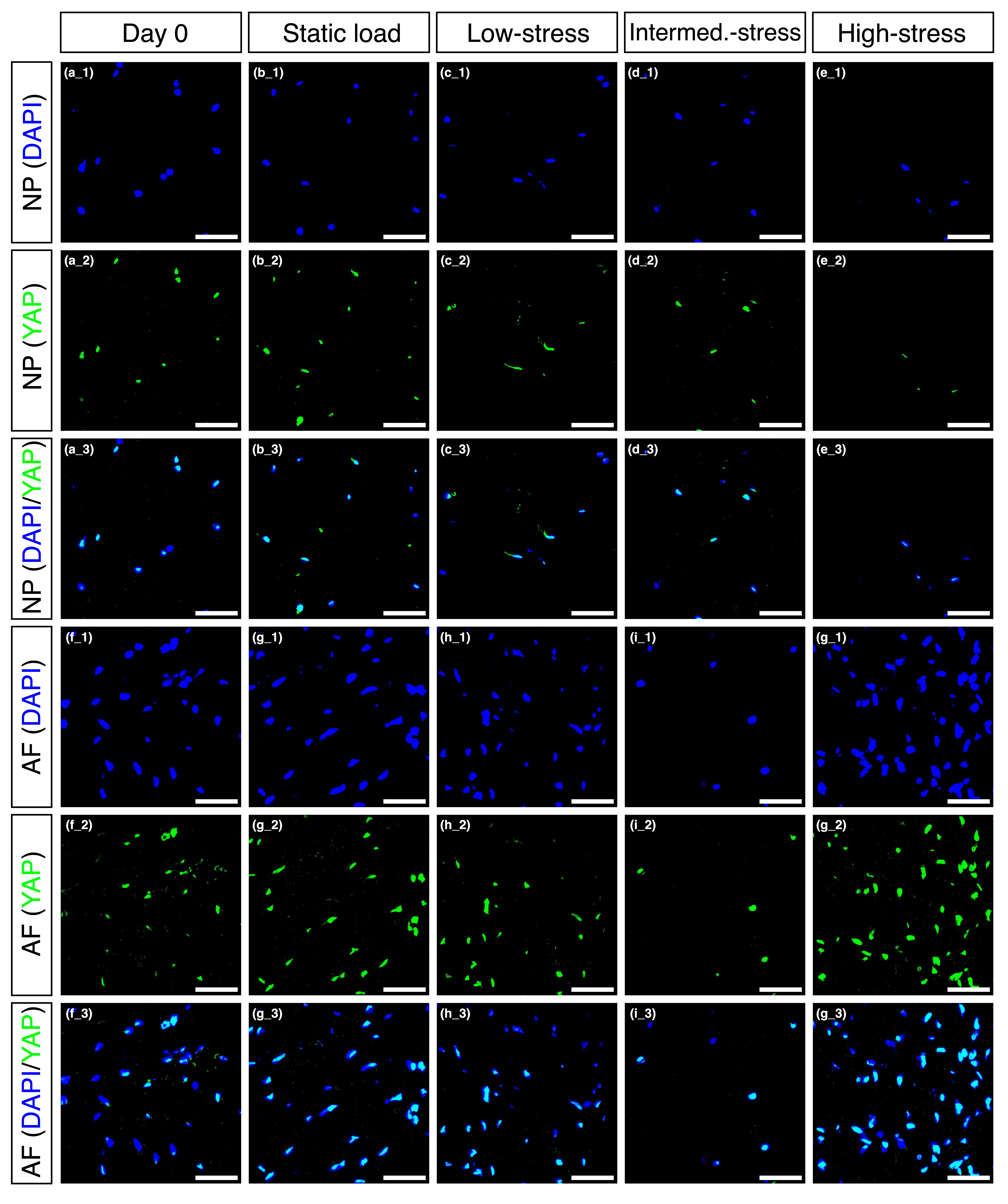

Supplement: Supplementary file 1 [file ijms-22-13641-s001.zip › Manuscript+Figures/Figure_S1.jpg]

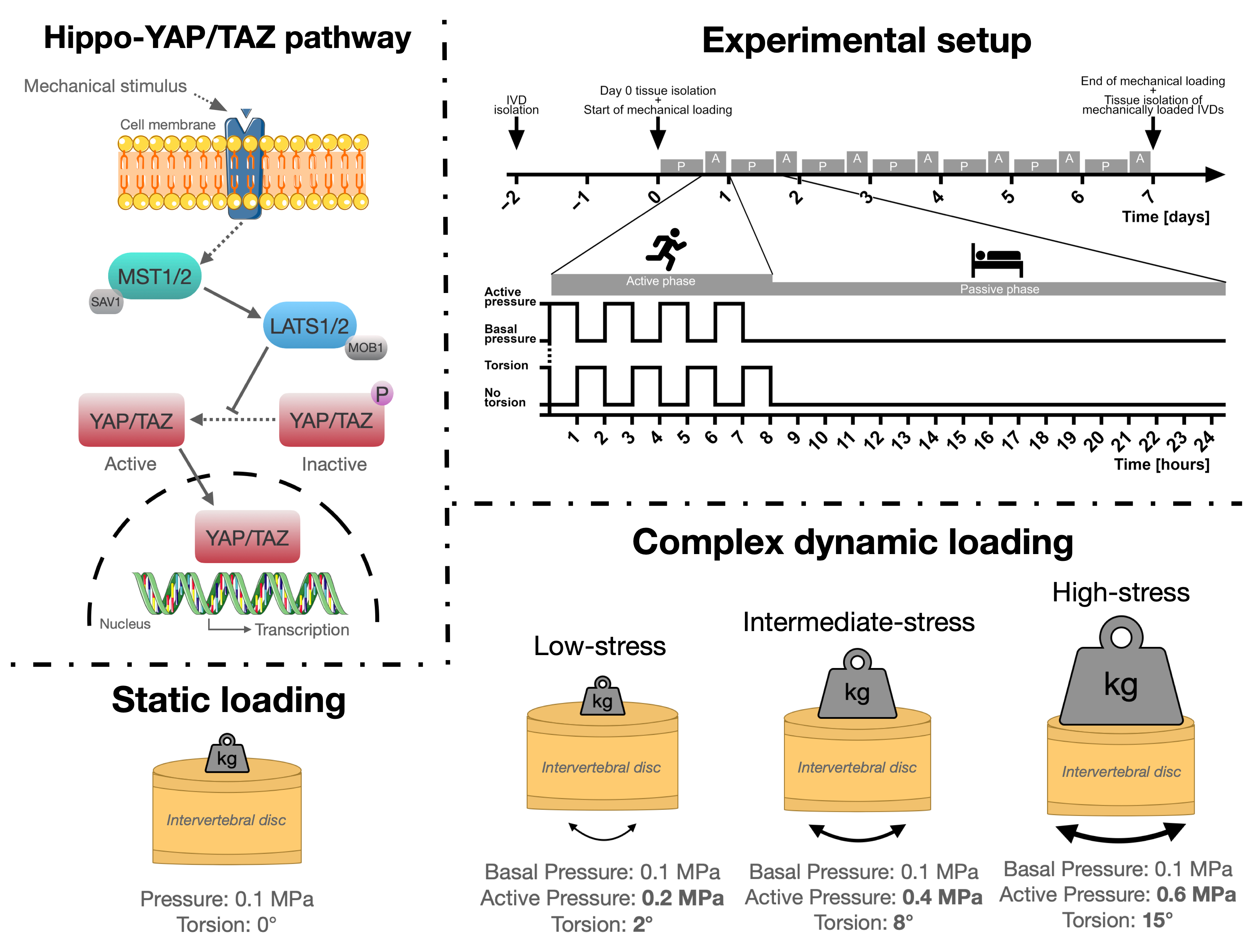

Supplement: Supplementary file 1 [file ijms-22-13641-s001.zip › Manuscript+Figures/Graphical abstract.jpg]
